# Accuracy and Resolution Analysis of a Direct Resistive Sensor Array to FPGA Interface

**DOI:** 10.3390/s16020181

**Published:** 2016-02-01

**Authors:** Óscar Oballe-Peinado, Fernando Vidal-Verdú, José A. Sánchez-Durán, Julián Castellanos-Ramos, José A. Hidalgo-López

**Affiliations:** 1 Departamento de Electrónica, Universidad de Málaga, Andalucía Tech, Campus de Teatinos, Málaga 29071, Spain; oballe@uma.es (O.O.-P.); jsd@uma.es (J.A.S.-D.); julian@elca.uma.es (J.C.-R.); jahidalgo@uma.es (J.A.H.-L.); 2 Instituto de Investigación Biomédica de Málaga (IBIMA), Málaga 29010, Spain

**Keywords:** resistive sensor arrays, direct sensor-to-digital device interface, FPGAs, parallel analogue data acquisition

## Abstract

Resistive sensor arrays are formed by a large number of individual sensors which are distributed in different ways. This paper proposes a direct connection between an FPGA and a resistive array distributed in *M* rows and *N* columns, without the need of analog-to-digital converters to obtain resistance values in the sensor and where the conditioning circuit is reduced to the use of a capacitor in each of the columns of the matrix. The circuit allows parallel measurements of the *N* resistors which form each of the rows of the array, eliminating the resistive crosstalk which is typical of these circuits. This is achieved by an addressing technique which does not require external elements to the FPGA. Although the typical resistive crosstalk between resistors which are measured simultaneously is eliminated, other elements that have an impact on the measurement of discharge times appear in the proposed architecture and, therefore, affect the uncertainty in resistance value measurements; these elements need to be studied. Finally, the performance of different calibration techniques is assessed experimentally on a discrete resistor array, obtaining for a new model of calibration, a maximum relative error of 0.066% in a range of resistor values which correspond to a tactile sensor.

## 1. Introduction

An important number of sensors are based on the variation of resistance values shown due to the passage of an electric current, depending on a physical magnitude which is measured. These are resistive sensors, which can be classified according to their applications. They can be tactile sensors [1,2,3,4,5,6,7,8,9,10], temperature sensors [11,12], gas detectors [13,14,15] or for other substances [16,17]. In order to obtain the desired information, sometimes a single sensor can be enough. Several sensors are necessary in other occasions. For example, tactile sensors used in applications such as skin emulation or robotic hands (where large surfaces need to be scanned) are usually formed by an array with a large number of individual sensors. Substance detectors are also usually formed by a large number of sensors.

One of the main problems of resistive sensor arrays is that they need a large number of components and complicated wiring for the circuits which measure the different resistances. There are also numerous circuits carrying out signal conditioning and analog-to-digital conversion of the information for its subsequent processing. In principle, for a single access (SA) configuration 2·*M*·*N* cables from the sensor to the circuit are needed, although one of them is normally shared by all sensors [10] so they are reduced to *M*·*N* + 1. The time needed to extract the information of the whole array is practically the time needed to extract the information of a single sensor. However, each sensor needs its own circuit for conditioning and AD conversion. For this reason, for large size arrays this configuration can be very costly in terms of area and energy consumption.

With the aim of alleviating this problem, a resistive sensor array can be distributed in the shape of a two-dimensional matrix of *M* rows and *N* columns. There are several sensors sharing the rows and columns in this array access (AA) configuration, but in such a way that the information of a single sensor can be accessed by selecting a row and a column. Therefore, the number of cables from the array is reduced to *M* + *N* and the number of necessary circuits to scan the information would be *M* or *N* depending on the type of array. In addition to this, additional circuits are needed to carry out sensor addressing. On the other hand, the time needed to extract the information of the whole array is the result of multiplying the time used in scanning a sensor by the number of *N* columns or *M* rows of the array plus the time required in multiplexing. This same solution has to be used if the construction of the sensor already has a structure of rows and columns and its output comes through *M* + *N* wires, as in [2,4]. A second possibility to further reduce the circuitry which carries out AD conversion is the increase of the multiplexing of the information of the array in such a way that only one element is scanned at a time. This solution is obviously slower and the multiplexing circuits are more complicated.

The last two strategies to scan a resistive sensor array show, in addition to the increase of the time needed to access the information, the so-called crosstalk effect [11,18,19,20,21,22,23]. This effect happens when the resistance values of other elements of the array have an impact on the measurement of the resistance of the element which is being scanned. Because of this effect, resistance measurements show a certain degree of error when the structures mentioned above are used. For instance, errors of 30% are obtained for a 4 × 4 array with resistors in a range between 100 Ω and 1000 Ω (using buffers with internal resistance of 10 Ω to address the rows) [24], despite the use of four operational amplifiers to reduce crosstalk. This error can be reduced with more complex circuits including calibration techniques to improve the readout accuracy [25] or including analog switches and an additional OA [24]. Therefore, for accurate and fast readings without these complex circuits, the only solution is accessing each of the elements of the array individually and without multiplexing.

The solution used in this paper avoids resistive crosstalk, providing accuracy in the measurement as in a SA configuration, while having the intermediate speed and intermediate hardware cost of an AA configuration. The structure of the array is divided into rows and columns, but now, in each column, all sensors share a terminal while the other is individual (row access configuration, RA). Therefore, the sensor would have (*M* + 1*)*·*N* cables and the information of *N* sensors will be accessed simultaneously. The signal conditioning circuit is only one capacitor in each column, which is also part of the circuit for AD conversion (for our case a time-to-digital conversion). This is carried out by using an FPGA as in [26] instead of microcontrollers [27]. This configuration, with some modifications, can be applied to other sensors (capacitive or inductive) that provide a time-modulated signal that can be directly measured in the digital domain [28,29].

Another reason to use this array configuration lies in the need of calibrating the measurement of resistive sensors. There is extensive literature on calibration of single sensors [26,30,31,32,33], but not on calibration of resistive sensor arrays. In general, calibration is necessary to avoid errors in the measurement of parameters which are used in the determination of resistance values. In an AA type sensor, for best accuracy, *M*·*N* calibration resistors would also be needed, as the values of the capacitors which are used by each resistor of the array have to be calibrated as well. Calibrating the threshold voltage which makes digital devices go from detecting a 1-logic to a 0-logic would also be necessary if microcontrollers or FPGAs are used for AD conversion. This greatly complicates the total hardware of the system. However, only *N* calibration elements are necessary if an array with an RA configuration is used.

FPGAs have a large number of input/output ports which can be connected directly to the array, avoiding the need of additional circuits. They also allow for parallel processing of the digital information obtained simultaneously from the *N* sensors. For this reason, a circuit with a direct sensor-FPGA connection seems to be the best option. This type of architecture was shown in [34], however, the effects on precision and accuracy of having *M* sensors sharing the conditioning circuit and AD conversion were not studied, or the limitations this imposes on the number of rows that the array can have, or the hardware requirements of the FPGA to carry out sensing functions.

A characterization of this configuration of the sensor which takes into account all the facts mentioned above is carried out in this paper. The results are obtained experimentally using a circuit which will simulate the operation of a tactile sensor array, from discrete known resistors. Finally, the accuracy of the circuit will be analyzed with several techniques.

The structure of this paper is as follows: Section 2 describes the architecture and the operation mode of the proposed circuit of direct connection with an RA configuration; Section 3 studies what we will refer to as *RC crosstalk*, which derives from the RA configuration of the circuit; Section 4 explains how to assess all components which have an impact on uncertainty in time measurement of the resistors of the matrix; Section 5 describes the materials and procedures used for the implementation of the circuit; Section 6 shows the experimental results obtained and analyses the consequences of using different calibration techniques to estimate resistance values in the array. The conclusions section closes the paper.

## 2. Description of Architecture and Operation Mode

**Figure 1:**
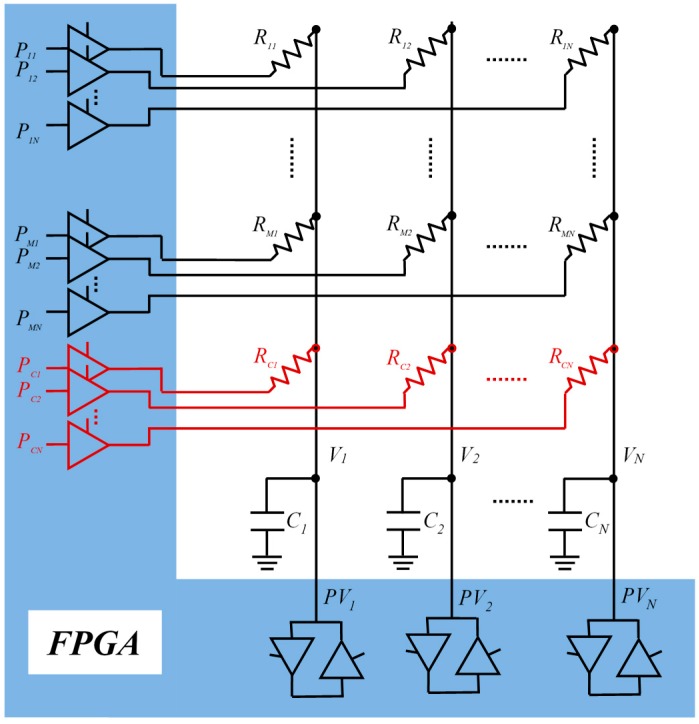
Direct interface resistive sensor array-FPGA.

Figure 1 shows the proposed architecture as a direct interface to a tactile sensor with an RA configuration. As can be observed, there is no physical multiplexing circuit outside of the FPGA. The number of pins needed in the FPGA to address the sensor array is (*M* + 1)·*N*. In the array, each *R_ij_* resistor is connected to a *P_ij_* pin in the FPGA which can be set as output (supplying voltages close to 0 V or to *V_DD_*) or in high impedance (HZ). The common cable to all sensors of a column is connected to a *C_j_* capacitor and to a *PV_j_* pin of the FPGA which can be set as input/output (I/O). Additionally, each column has an extra *R_cj_* resistor (not a sensor) connected to the *P_cj_* pin, which is used for calibration purposes, as described further in this paper. Obviously, the maximum size of the sensor will be determined by the number of pins available in the FPGA, although this is not currently a severe limitation, as FPGAs with ranges of hundreds of pins can be found for use [35].

The reading process of each row of the array has two phases. Firstly, in the charge phase, *C_j_* capacitors are charged by setting *PV_j_* pins as output to a ‘1’ level while all *P_ij_* pins are kept in HZ. Then, in the discharge phase, a whole row is selected by setting its corresponding I/O pins to ‘0’. For instance, pins *P_k1_*, …*P_kN_* are set to ‘0’ while the remaining *P_ij_* pins, are kept in HZ. Simultaneously, all *PV_j_* pins are selected as input to be able to measure the *V_j_* voltage of the *C_j_* capacitor which is being discharged. A set of timers are started in the FPGA at the beginning of this discharging phase and their counts are stopped when the low threshold (*VT_j_*) is reached at the related column pins. *VT_j_* is the voltage in which the FPGA detects a ‘0’ logic in *PV_j_*. Therefore, a whole row is read in parallel. The process is repeated for the M rows of the array and the calibration row.

The purpose of the calibration resistor is to avoid having to know the *C_j_*, *VT_j_* and *V_DD_* values, because these are normally difficult to measure. In addition, *C_j_* and *VT_j_* depend on power voltage supply or temperature and they can drift with time. As there is only one calibration resistor, it would seem that the method used is what is known in the literature as a single-point calibration [27]. According to this method, if when row i is selected, the discharge time measured for the *C_j_* capacitor is Δ*t_ij_*, and when the calibration row is selected, the time is Δ*t_cj_*, then the *R_ij_* resistance value is determined by:

(1) $$R_{ij}=\frac{\Delta t_{ij}}{\Delta t_{cj}}R_{cj}$$

where *R_cj_* is a value decided by the designer. However, this calibration method does not take into account the output resistance *RB_j_* of the FPGA buffers (around 10 Ω for the FPGA of this paper) which are connected in series to *R_ij_* and *R_cj_*. For this reason, if Equation (1) is used, a mistake is made in the estimation of resistance value. More accuracy could be obtained if the two-point calibration method is used [27]. However, adding an additional row of calibration is then necessary, resulting in an increase of hardware in the circuit.

A calibration solution which takes into account *RB_i_* resistances, and uses a single calibration resistor is proposed in [36]. The method consists on using off-time calibration (outside of the normal operation of the sensor) where, by using different resistors and measuring their discharge time, a relationship can be established between *R_ij_* and *R_cj_*. It is shown that the adjustment of the experimental data using a linear function is sufficiently accurate, resulting in the following relationship:

(2) $$R_{ij}=\alpha \cdot \Delta t_{ij}^{\prime }+B$$

with *α* and *B* as constants and where $\Delta t_{ij}^{\prime }$ would be the time used for discharging *C_j_* through *R_ij_* during off-time calibration. $\Delta t_{cj}^{\prime }$ must also be measured during off-time calibration. This is the time used in the discharge through *R_cj_* (calibration resistor which will be used during the normal operation of the sensor). By using this time value, Equation (2) *R_ij_* can be calculated as follows:

(3) $$R_{ij}=A\cdot \frac{\Delta t_{ij}^{\prime }}{\Delta t_{cj}^{\prime }}+B$$

where *A* = *R_cj_* − *B*. Using Equation (3), during the normal operation of the circuit, the value of *R_ij_* can be calculated by measuring the Δ*t_ij_* and Δ*t_cj_* times:

(4) $$R_{ij}=A\cdot \frac{\Delta t_{ij}}{\Delta t_{cj}}+B$$

This way of obtaining *R_ij_* is similar to that of a single-point calibration but taking the *RB_i_* buffer resistances into account and, therefore, with an accuracy which is similar to that of a two-point calibration. We will refer to this procedure as the *off-on time* method. This technique has the drawback of requiring prior pre-calibration, although the hardware is simpler, so the cost is reduced. Moreover, the time required to scan the array decreases when compared with that of the two-point calibration method, and the power consumption is also lower.

Although in principle there is no crosstalk in Equation (4), it must be noted that this expression does not take stray capacitors which appear in the FPGA pins into account, which are in HZ state when an array row is read. This originates what will be referred to as *RC crosstalk* in this paper, which will be studied in detail in the following section.

## 3. RC Crosstalk

**Figure 2:**
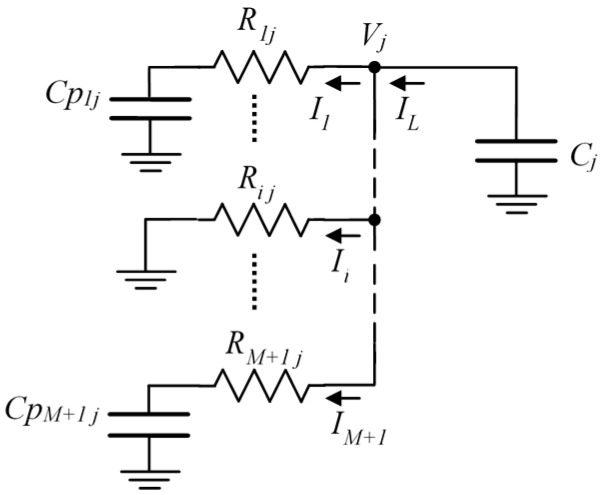
Detail of the *j* column of the circuit, showing the stray capacitors.

Figure 2 shows the detail of the *j* column of the circuit when the discharge of *C_j_* is being measured through the *R_ij_* resistor. *Cp_kj_* with k≠i are the capacitors which group all stray capacitors connected to *P_ij_* pins in HZ state in the FPGA. By analyzing Figure 2, it can be observed how all stray capacitors have an impact on the discharge process of *C_j_*. Moreover, the impact is not always the same, as the resistors which link them to *C_j_* also vary. In Figure 2, it has been assumed that the discharge of the *Cp_ij_* capacitor is very fast compared to that of C*_j_*, (and it is true, as *RB_i_* << *R_ij_* and *Cp_ij_* << *C_j_*).

*V_j_*(*s*) is the voltage of *C_j_* in the Laplace variable, during the discharge process. Its value is determined by:

(5) $$V_{j}(s)=\frac{-I_{L}}{C_{j}s}+\frac{V_{DD}}{s}$$

where *I_L_* is the current which discharges the capacitor and *V_DD_* is its initial charging voltage. On the other hand, for each *I_K_* with *k* ≠ *i*:

(6) $$V_{j}(s)=I_{k}\left(R_{k\;j}+\frac{1}{Cp_{k\;j}}\right)+\frac{V_{DD}}{s}$$

while:

(7) $$I_{i}=\frac{V_{j}(s)}{R_{ij}}$$

Solving for *I_L_* in Equation (5), *I_k_* in Equation (6) and taking into account that:

(8) $$I_{L}=\sum_{k=1}^{M+1}I_{k}$$

where *k = M +* 1 indicates the calibration row, the equation is:

(9) $$-V_{j}(s)C_{j}s+C_{j}V_{DD}=\frac{V_{j}(s)}{R_{ij}}+\sum_{\begin{array}{l} k=1\\ k\neq i \end{array}}^{M+1}\frac{1}{R_{k\;j}}\frac{sV_{j}(s)-V_{DD}}{s+\frac{1}{R_{k\;j}Cp_{k\;j}}}$$

solving for *V_j_*(*s*):

(10) $$V_{j}(s)=V_{DD}\cdot \frac{C_{j}+\sum_{\begin{array}{l} k=1\\ k\neq i \end{array}}^{M+1}\frac{1}{R_{k\;j}}\frac{1}{s+\frac{1}{R_{k\;j}Cp_{k\;j}}}}{C_{j}s+\frac{1}{R_{i\;j}}+\sum_{\begin{array}{l} k=1\\ k\neq i \end{array}}^{M+1}\frac{1}{R_{k\;j}}\frac{1}{s+\frac{1}{R_{k\;j}Cp_{k\;j}}}}$$

and finally:

(11) $$V_{j}(s)=V_{DD}\cdot \frac{1+\sum_{\begin{array}{l} k=1\\ k\neq i \end{array}}^{M+1}\frac{Cp_{k\;j}}{C_{j}}\frac{1}{R_{k\;j}Cp_{k\;j}s+1}}{s\left(1+\sum_{\begin{array}{l} k=1\\ k\neq i \end{array}}^{M+1}\frac{Cp_{k\;j}}{C_{j}}\frac{1}{R_{k\;j}Cp_{k\;j}s+1}\right)+\frac{1}{R_{i\;j}C_{j}}}$$

Some facts are clear from Equation (11). Firstly, *V_j_*(*s*) is not only the discharge voltage of a capacitor with a single pole; it has *M* + 1 poles and *M* zeros. Equation (11) also shows crosstalk appearance, as *V_j_*(*s*) is not only a function of *R_ij_* and *C_j_*; it also depends on the rest of the resistors of the column (*R_kj_*) and on the *Cp_kj_* stray capacitors associated to each pin in the column (this is where the name RC crosstalk comes from).

Secondly, if *Cp_kj_* << *C_j_*, Equation (11) is reduced to:

(12) $$V_{j}(s)\approx \frac{V_{DD}}{s+\frac{1}{R_{i\;j}C_{j}}}$$

which is the discharge equation through an only capacitor. Therefore, the higher *C_j_* is, compared to *Cp_kj_*, the lower the crosstalk will be. Reducing *Cp_kj_* implies making a good design of the sensor and of the control circuit. However, there are terms composing *Cp_kj_* which cannot be reduced, such as FPGA pin capacitance or that of their contacts with the wiring of the sensors (once the PCB technology of the sensors has been selected). The dependency with *R_kj_* can also be observed in Equation (11). But this term cannot be varied as it is the resistance of the sensors themselves.

**Figure 3:**
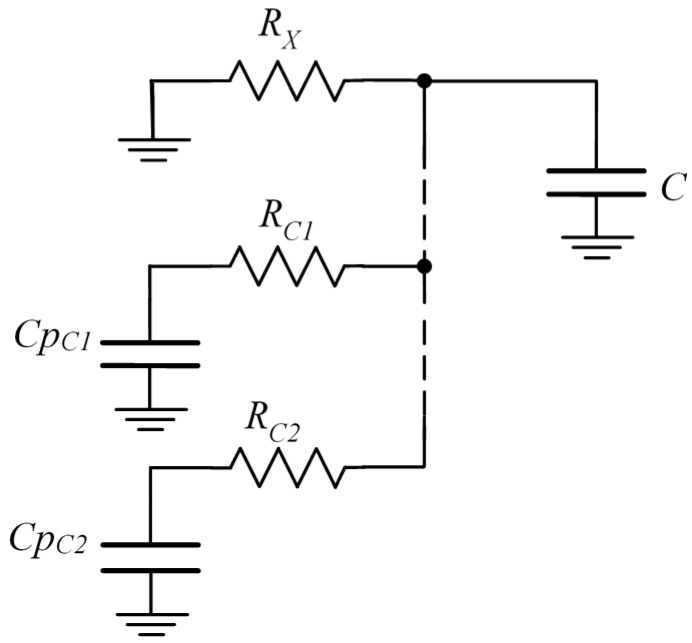
Two-point calibration circuit showing RC crosstalk due to stray capacitors.

Although RC crosstalk has been analyzed in a sensor array, it will also appear if any of the calibration techniques mentioned in Section 2 are used. This situation occurs even when a single sensor is calibrated. Figure 3 shows a circuit for the measurement of an *R_X_* resistor, with two calibration resistors, *R_C1_* and *R_C2_*. As shown in the figure, *R_C1_* and *R_C2_* resistors are associated with two parasitic capacitors, *Cp_C1_* and *Cp_C2_*.

## 4. Measurement Uncertainty Analysis

Equation (11) also helps us find the maximum Δ*t*max*_ij_* and the minimum Δ*t*min*_ij_* times used by the FPGA to detect a 0-logic in the discharge process through *R_ij_*. Then, if *R_kj_* tends to 0, Equation (11) would be:

(13) $$V_{j}(s)=\frac{V_{DD}}{s+\frac{1}{R_{i\;j}C_{eq}}}$$

with:

(14) $$Ceq_{ij}=C_{j}+\sum_{\begin{array}{l} k=1\\ k\neq i \end{array}}^{M+1}Cp_{kj}$$

On the other hand, if *R_kj_* → ∞ then, we find again that Equation (11) transforms into Equation (12). Then, the time needed to discharge *C_j_* at a *VT_j_* voltage using Equation (11) is limited by discharge times at that same voltage of two RC circuits with capacitors of different values and the same resistor. This is:

(15) $$\Delta t\max_{ij}=R_{ij}Ceq_{ij}\cdot \ln\left(\frac{VT_{j}}{V_{DD}}\right)$$

(16) $$\Delta t\min_{ij}=R_{ij}C_{j}\cdot \ln\left(\frac{VT_{j}}{V_{DD}}\right)$$

Therefore, Δ*t*max*_ij_* < Δ*t_ij_* < Δ*t*mix*_ij_* and, its exact value will depend on the rest of the resistors. 

Another way of interpreting these results involves considering Δ*t_ij_* as a statistical variable with its corresponding probability density function and standard deviation, σ(Δ*t_ij_*). Depending on the process followed to find the value of Equations (15) and (16), the probability density function of Δ*t_ij_* will depend on the range of values of the *R_kj_* resistors, in such a way that the smaller the range they can vary, the smaller σ(Δ*t_ij_*) will be. On the other hand, the standard deviation value will also be smaller, the smaller the difference between *Ceq_ij_* and *C_j_* is, or, in other words, the smaller the sum of the values of *Cp_kj_* is. But, as has been mentioned earlier, this has a technological limitation which imposes a minimum value to σ(Δ*t_ij_*). It can also be observed how an increase in the number of *M* rows in the array, increases the value of the sum of *Cp_kj_*, and therefore of σ(Δ*t_ij_*). If what we are interested in is the relative standard deviation, σ(Δ*t_ij_*)/Δ*t_ij_*, this is, according to Equations (15) and (16), a function of *Ceq_ij_/C_j_* and of the probability density function of the resistances. Therefore, a possibility to reduce this, is the increase of *C_j_*, but this is inconsistent with the sensing speed of the array. Consequently, there is a tradeoff between speed and accuracy in the measuring of resistances. This same effect also appears in the case of a sensor with only one component, where an increase of the capacitor to be discharged also implies an increase in accuracy, in this case, related to the reduction of the error term due to quantification [27]. Obviously, this quantification error also appears in the measures of the resistances of the array. Therefore, and in sum, the measuring of the Δ*t_ij_* time, corresponding to a specific resistor of the array, has as uncertainty resources related to other sensors: the values of the other resistors of the array and the existence of *Cp_kj_* capacitors. This causes a first source of uncertainty, crosstalk uncertainty, σ*_crosstalk_*(Δ*t_ij_*). The other sources of uncertainty that a single sensor which is not part of an array would have, must be added to this: *u(z)* quantification noise and the noise in *V_DD_*, *VT_j_* and *C_j_*, which generate what we will refer to as trigger noise uncertainty, σ*_trigger_*(Δ*t_ij_*).

The same procedure can be followed if the aim is to measure the time needed to get a 0-logic for the calibration resistor (Δ*t_cj_*). This time value will also be a random variable with extreme values determined by:

(17) $$\Delta t\max_{cj}=R_{cj}Ceq_{cj}\cdot \ln\left(\frac{VT_{j}}{V_{DD}}\right)$$

(18) $$\Delta t\min_{cj}=R_{cj}C_{j}\cdot \ln\left(\frac{VT_{j}}{V_{DD}}\right)$$

where:

(19) $$Ceq_{cj}=C_{j}+\sum_{k=1}^{M}Cp_{k\;j}$$

and therefore, with its own standard deviation, σ(Δ*t_cj_*). It must be noted that the differences between the Δ*t_ij_* and Δ*t_cj_* values are not only determined by the different *R_ij_* and *R_cj_* resistance values, but also because, in general, *Ceq_cj_* ≠ *Ceq_ij_* with *i* ≠ *c*, due to the different capacitors associated to the wiring which links each sensor with its corresponding pin in the FPGA (these differences can become very small with a careful design and adequate technology, although they will always exist). This also shows that if the position of the resistors in a column was changed (with the exception of that for calibration) variations in their values would appear. Then, as it has been shown with *R_cj_*, there is a third source of uncertainty associated to the difference between the capacitances of the nodes in a column. This will originate a standard deviation in time measurement which will be referred to as σ*_column_*(Δ*t_ij_*).

It could also be argued that there is noise due to the position in the same row but in different columns. However, assuming that the distribution of resistance values is independent of the rows and that the variation in the *Cp_ij_* capacitors is identical in all columns, this noise would only be due to the variations in *C_j_*. This is compensated through the calibration circuit of each column, so it is considered as insignificant. Therefore, uncertainty in Δ*t_ij_* measurement, can be expressed by:

(20) $$\sigma \left(\Delta t_{ij}\right)=\sqrt{\sigma_{crosstalk}^{2}\left(\Delta t_{ij}\right)+\sigma_{trigger}^{2}\left(\Delta t_{ij}\right)+\sigma_{column}^{2}\left(\Delta t_{ij}\right)}$$

as the three terms inside the root are independent of each other.

Noise due to quantification, $u(z)=T_{s}/\sqrt{12}$, should be added to this deviation [37], where *T_s_* is the period of time of the counter which measures discharge time. Although uncertainty in *V_DD_*, *VT_j_* and *C_j_* values can be compensated using Equation (4) (reducing then the values of all terms inside the root), there is still uncertainty in measurement due to RC crosstalk. According to Equations (15) and (16), there are three possibilities to reduce the relative impact of crosstalk: the first one is in the increase of *C_j_*, but, as we have mentioned before, this reduces the scanning speed of the array. The second possibility is the reduction of the *Cp_ij_* terms, but this (once a careful design of the system has been carried out) is limited by technology and the number of rows in the array. The third possibility is the limitation in the range of values of the resistors of the array, but obviously, this can only be achieved in some very specific sensors. Therefore, although the RA structure of the sensor array does not show the typical resistive crosstalk, there exists what has been referred to as RC crosstalk and which, therefore, has to be assessed.

### Assessment Method of Uncertainty in Measurement

A naive method for uncertainty assessment in Δ*t_ij_* measurement would be to perform a set of measurements for each of the sensors of the array, varying the resistors of the remaining sensors in each of the measurements, obtaining then the maximum ranges of variation. However, the use of this method of characterization is a difficult task to undertake, as the number of sensors in an array is not usually a small number, even in our case where a “reduced” 7 × 8 sensor array has been used.

The aim of this paper is to propose the assessment of total uncertainty in the measurement of any resistor of the array based on the independency of uncertainties due to crosstalk, column and trigger by using Equation (20).

For this reason, a circuit simulating by means of resistors the different values which can be read from all positions of an array with RA configuration has been designed. The circuit, obviously, includes an FPGA and the necessary capacitors for time-to-digital conversion.

The assessment method of uncertainty in measurement comprises the following steps: firstly, a set of sufficient resistor values which approximately divide the range of the sensor in uniform sections is selected (8 in our case). Each of these resistors is placed in successive tests in the *M* rows of a column of the array selected for characterization. The remaining resistors of the column take the mean value in the range. Then, a set of *Q* time measurements are carried out (Δ*t_ij_*). The process is repeated *M* times changing the position of the resistor in a circular manner, in such a way that *M*·*Q* time measures are obtained for each resistor. This set of times has a standard deviation which is due both to trigger uncertainty and to position uncertainty in the column, with variance $\sigma_{trigger+column}^{2}$.

To get the value of each of these terms $\sigma_{trigger}^{2}$ and $\sigma_{column}^{2}$ (for each resistor), the following procedure is proposed. From a set of *Q* measurements carried out in each row, *M* $\sigma_{trigger}^{2}$ values are obtained. These values should be very similar for all rows, and the mean of these values is the final estimation of $\sigma_{trigger}^{2}$. Because they are independent sources of uncertainty, its variance is $\sigma_{trigger\;+\;column}^{2}=\sigma_{trigger}^{2}+\sigma_{\;column}^{2}$ and therefore $\sigma_{column}^{2}$ could be calculated as:

(21) $$\sigma_{column}^{2}=\sigma_{trigger\;+\;column}^{2}-\sigma_{trigger}^{2}$$

σ*_crosstalk_* can be found with the following procedure: a row in the column used for the set of previous measurements is selected. Each of the eight resistors will be placed in this position of the array. Two sets of *Q* measurements will be carried out for each resistor. In the first set of *Q* measurements, the minimum value resistor in the sensor is added to the selected resistor in the remaining positions of the column. In the second set of *Q* measurements, minimum value resistors are replaced by those with maximum values. Then we find the Δ*tmax* maximum and Δ*tmin* minimum time values for the 2·*Q* measures, and their difference Δ*tmax* − Δ*tmin*, will be the estimator used to find the range of Δ*t_ij_* values for each resistor due to RC crosstalk. Finally, assuming that the values of the sensor resistors have a uniform distribution and, therefore, Δ*t_ij_* as well, for each resistor, the σ*_crosstalk_* value can be calculated as:

(22) $$\sigma_{trigger+crosstalk}=\frac{\Delta t\max-\Delta t\min}{\sqrt{12}}$$

where again we can solve for σ*_crosstalk_*:

(23) $$\sigma_{crosstalk}^{2}=\sigma_{trigger+crosstalk}^{2}-\sigma_{trigger}^{2}$$

Therefore, the value of σ(Δ*t_ij_*) can be obtained for each resistor by using Equation (20). Quantification uncertainty, *u*(*z*), should be added to this, obtaining then a total uncertainty value, σ*_T_* (Δ*t_ij_*), determined by:

(24) $$\sigma_{T}\left(\Delta t_{ij}\right)=\sqrt{\sigma^{2}\left(\Delta t_{ij}\right)+u^{2}(z)}$$

Uncertainty in the whole range will be the maximum value of all uncertainties obtained.

## 5. Materials and Methods

**Figure 4:**
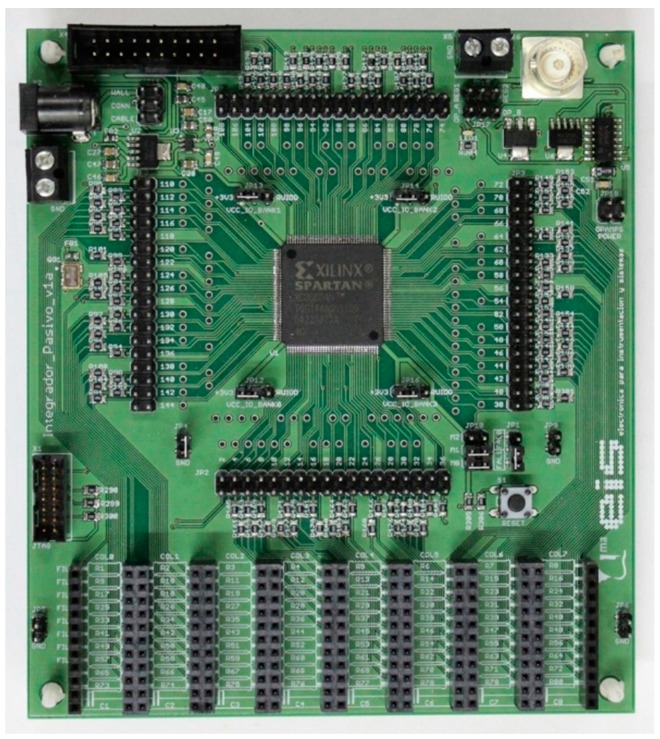
Setup to test the direct interface circuit for resistive sensor array.

The circuit in Figure 4 follows the diagram proposed in Figure 1. It has been made using a Xilinx Spartan3AN FPGA (XC3S50AN-4TQG144C) [35] and an oscillator circuit based on crystal quartz with an operating frequency of 50 MHz. Each of the eight capture modules has a 14-bit counter, with a 20 ns time base. The maximum current that an output buffer of the FPGA can sunk for maintaining the digital signal integrity of the outputs is 24 mA. For currents above 24 mA the output voltage of the buffer that drives the row is higher than that identified as a ‘0’ logical value, the output transistor is not in the ohmic region and the resistance of the buffer *RB*_j_ changes significantly. However, it is possible to add a resistance in series with the one that is measured to limit the current and this does not alter the procedure described above.

The design rules recommended by the manufactures of the FPGA are applied rigorously so that noise impact affecting *VT_j_* causes the smallest uncertainty possible in measurement. This FPGA works with independent supply voltages for the input/output blocks and the digital processing core where the rest of the circuitry resides. The use of two independent voltage regulators is then necessary in order to reduce the influence of the activity of the core in the input/output buffers. The selected regulators, TPS79633 [38] for the voltage of the input/output banks supplied at 3.3 V and TPS79912 [39] for the voltage of the core at 1.2 V, have extremely low values in their output voltage, both for dropout voltage during maximum charge as well as for output voltage noise (40 µV RMS). Also, they only need a few external components for correct operation, which make them ideal for devices where the area occupied by the circuit is large. For each of the four buffer banks included in the FPGA, a battery of decoupling capacitors of different values in a position very close to the supply inputs are used. These are connected through two supply planes; the first one for the 3.3 V voltage and the other for GND. The printed circuit board where the circuit is mounted on has been manufactured with a FR-4 fiberglass substrate and four layers, leaving internal layers for supply planes and external layers for the remaining signals.

The resistor array to be measured is composed of ten rows by eight columns with an RA structure. As has been mentioned, the RA structure makes columns electrically independent between them; therefore the experimental tests are focused on the study of a single column and the ten resistors which can be measured through an only input port, although the design has been made to allow for 80 resistors to be placed.

As indicated earlier, the sensor to be modeled is composed of seven rows and eight columns. Three additional rows have been added in order to asses different calibration methods. Two of them will then be used for a two-point calibration, which will have resistors placed approximately within 15% and 85% of the range where measuring is to be carried out [40]. The third row will use a resistor in approximately 50% of the range which will be used either for a single-point calibration or for the *off-on time* method, using Equation (4).

**Figure 5:**
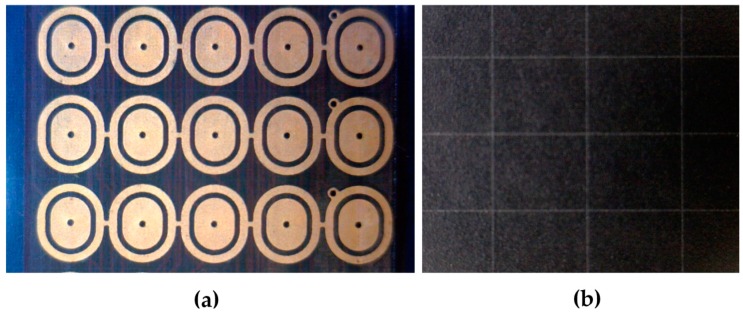
Details of the electrode matrix of the finger sensor (**a**) and of the discrete material (**b**).

Figure 5a shows a detail of the implemented electrodes array which is going to be simulated with discrete resistors. Each sensor is connected to a solid and oval inner electrode through which the terminal of the sensor is accessed individually. On the other hand, the outer electrodes enclose the inner electrodes. It can be observed how all outer electrodes in a column (horizontally in the image) are connected between them, forming the common terminal of the column. Electrodes are placed in a PCB with “Rigiflex” technology of four layers and an IPC-6013 reference standard [41]. 

Figure 5b shows the sheet of discrete piezoresistive material (divided into rectangular sections) which is placed on the electrode matrix. When pressing the sheet, the resistance between the inner and outer electrodes varies. The sheet has been developed by CIDETEC and the range or resistor values can vary approximately between 7400 Ω for pressures of a few kPa to a few dozens of ohms for high pressures.

Experimental tests are performed with 8 resistor values within this range. The resistors used are: 199.96 Ω, 1297.73 Ω, 2401.95 Ω, 3687.49 Ω, 4836.09 Ω, 5810.79 Ω, 6966.87 Ω and 7348.84 Ω (which includes the resistance value of approximately 50% of the range, 3687.49 Ω). As for the capacitor, one with a 47 nF nominal value is selected, which complies with the design rules proposed in [33] for the constant of optimal time which minimizes relative standard uncertainty in measurement. Also, with a capacitor with this value, the sum of time values in the input/output cycles for the ten rows of the matrix allows reaching sampling rates which are higher than 200 tactile frames per second. The power consumptions of the FPGA without connecting the sensor array is 78 mA @ 50 MHz and it increases at a rate of 1 mA per column for a capacitor of 47 nF in our example implementation.

## 6. Results and Discussion

The aim of this section is to perform uncertainty and accuracy assessment in the measurement of resistance values in the circuit shown in the previous section. For this, the methodology proposed in Section 3 with *Q* = 500 will be followed. Table 1 shows the results for uncertainty obtained with the eight resistors used, where the indications proposed in [36] have been followed to design the capture module.

**Table 1:** Precision data for test resistors with a 47 nF capacitor and *Q* = 500.

Resistor (Ω)	σ*_trigger_*_+*column*_ (ns)	σ*_trigger_* (ns)	σ*_column_* (ns)	σ*_trigger_*_+*crosstalk*_ (ns)	σ*_crosstalk_* (ns)	σ (Δ*t_ij_*) (ns)	σ*_T_* (Δ*t_ij_*) (ns)
199.96	13.58	1.62	13.49	2.89	2.39	13.79	14.95
1297.73	18.06	5.24	17.29	11.55	10.29	20.79	21.58
2401.95	18.58	7.93	16.81	25.98	24.74	30.94	31.48
3687.49	29.83	11.49	27.53	30.31	28.05	40.95	41.35
4836.09	32.65	14.29	29.36	43.30	40.87	52.31	52.63
5810.79	40.13	17.77	35.98	56.29	53.41	66.81	67.06
6966.87	38.56	19.98	32.98	59.18	55.70	67.75	67.99
7348.84	38.46	21.42	31.94	70.73	67.41	77.60	77.82

The effective number of bits in time measuring (ENOB) obtained from σ*_T_* is 10.14 bits. To analyze the loss of precision due to the increase in the number of rows of the array, σ*_T_* has been measured for a situation in which there is only one resistor to be measured, as well as those for calibration. Table 2 shows a comparison between the results obtained and those of Table 1 for the resistor showing the worst results in both cases, 7348.84 Ω.

**Table 2:** Precision degradation due to increasing number of rows.

Rows (*M*)	σ(Δ*t_ij_*) (ns)	ENOB (bits)	Resolution (Ω)
1	21.40	12.04	1.70
7	77.82	10.14	6.34

As for accuracy in the estimation of resistance values, the results obtained with the traditional single-point and two-point calibration methods are going to be compared with those of the *off-on time* method indicated in Section 2. 

In order to carry out this comparison, as mentioned in Section 5, three additional rows were added for the different calibration methods. In these rows, resistors with 15% (1300 Ω), 85% (5820 Ω) and 50% (3680 Ω) values are placed. Therefore, we can assess all calibration methods in the same test.

For the *off-on time* method, the calibration curve has to be assessed first. This is done by placing the eight resistors shown in Table 1 in the same position. Two sets of tests are performed for each resistor, 500 measurements with the remaining resistors in the column with minimum values and another 500 with the resistors with maximum values. A linear adjustment is carried out from these 8000 measures [36]. Therefore, coefficients *A* and *B* of Equation (4) are obtained.

The same tests have then been performed for the three methods. Over a set of 14 resistor values which divide the possible range into approximately equal sections, 500 measurements are carried out with the remaining resistors with minimum values (Rmin = 199.96 Ω), 500 with resistors with medium values (Rmed = 3687.49 Ω) and 500 with resistors with maximum values (Rmax = 7348.84 Ω). 

Table 3 shows maximum absolute error values obtained from each of the three groups of 500 measurements. The last two rows show maximum absolute and relative errors in any circumstance.

**Table 3:** Accuracy data for different calibration techniques with a 47 nF capacitor and *Q* = 500.

	Maximum Absolute Error (Ω)								
Resistor (Ω)	Rmin (199.96 Ω)			Rmed (3687.49 Ω)			Rmax (7348.84 Ω)		
	1 P.	2 P.	Off-On	1 P.	2 P.	Off-On	1 P.	2 P.	Off-On
199.96	13.66	0.88	0.62	13.74	0.90	0.37	13.60	0.77	0.56
763.34	11.70	0.98	0.59	11.59	0.75	0.69	11.44	0.75	0.82
1297.32	10.03	0.65	0.58	10.11	0.76	0.67	9.79	0.65	0.62
1887.55	8.23	1.18	1.22	8.12	1.20	1.11	7.65	1.09	0.63
2401.95	5.69	0.81	0.84	5.42	1.18	1.08	4.98	0.74	1.44
3070.25	4.24	1.93	2.10	3.83	1.81	1.69	3.34	1.81	1.19
3684.25	1.83	2.21	2.21	1.60	1.94	1.98	1.13	2.07	1.51
4083.85	2.04	2.93	3.02	2.31	2.22	1.91	3.13	1.85	1.12
4836.05	5.73	2.19	2.46	5.68	2.32	1.88	6.67	2.02	1.56
5269.05	6.65	3.31	3.49	6.84	3.31	3.12	7.79	3.23	1.89
5813.45	9.32	2.64	2.72	9.88	2.65	2.37	10.63	2.88	1.68
6373.15	12.09	3.07	3.20	12.61	2.95	2.77	13.34	2.80	1.91
6983.15	16.29	2.29	2.35	15.75	2.37	1.81	17.34	2.69	3.41
7349.15	16.52	3.34	3.16	16.55	3.30	2.54	18.86	3.24	3.42
Total Error	124.03	28.39	28.56	124.03	27.67	23.99	129.69	26.59	21.77
Max. Absolute Error	16.52	3.34	3.49	16.55	3.31	3.12	18.86	3.24	3.42
Max. Relative Error (%)	0.225	0.045	0.066	0.225	0.063	0.059	0.257	0.044	0.047

**Figure 6:**
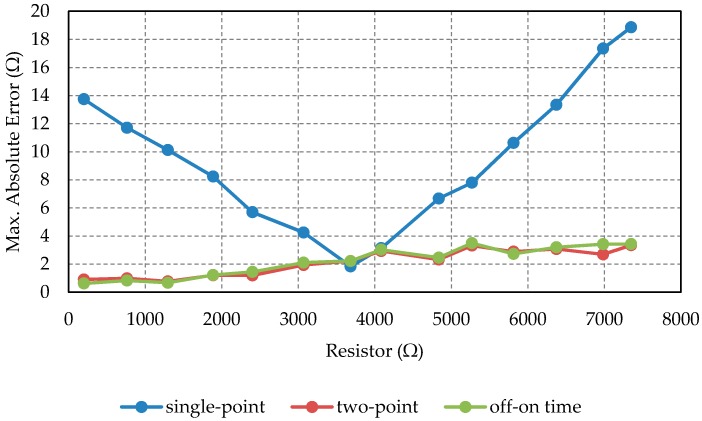
Worst absolute error for each resistor and calibration method.

As shown in Table 3, the errors for the two-point calibration and the *on-off time* calibration methods show very similar values. On the other hand, as expected, the errors for the single-point method are of a higher order of magnitude. As the *on-off time* method uses only one calibration resistor, it seems to be a better solution, because it uses simpler hardware and less time is needed to scan the whole matrix. Figure 6 shows the maximum absolute error for any condition using the three calibration methods.

## 7. Conclusions

This paper presents a direct connection circuit between an FPGA and a resistive sensor array which allows for parallel measuring of certain resistors of the array (a whole row). The addressing technique used (RA configuration) avoids the typical resistive crosstalk between the resistors of the array. For this, an input/output pin is used in each of the electrodes in the matrix. Although crosstalk is then avoided between resistors measured in parallel (those from one same row), there is another element, RC crosstalk, which affects uncertainty when measuring discharge time in capacitors (and which is used to estimate resistance) causing a reduction in the effective number of bits in time-digital conversion (ENOB).

The implementation of a circuit to perform a set of tests has been carried out. These tests simulate, with discrete resistors of known values, the behavior of a specific tactile sensor. The range of resistor values which has been assessed goes from 200 Ω to 7350 Ω.

In this case, the total uncertainty which includes all studied components (σ*_trigger_*, σ*_crosstalk_* and σ*_column_*) and also quantification noise, results in an ENOB of 10.14 bits. Besides the precision in the estimation of the resistance values, a study of accuracy has been performed by using different calibration techniques avoiding having to know certain values which are difficult to measure (*C_j_*, *VT_j_* and *V_DD_*), which could change with time and temperature.

From the calibration methods analyzed, the *off-on time* method, based on an adjustment to a linear function from data obtained in an operation mode previous to the normal mode, provides similar results to those of the two-point calibration technique, but only with the need of a reference resistor. Therefore both the hardware and the sampling time of the whole matrix are reduced. The maximum relative error obtained with this method has been 0.066%
